# Observed Thermal Impacts of Wind Farms Over Northern Illinois 

**DOI:** 10.3390/s150714981

**Published:** 2015-06-25

**Authors:** Lauren M. Slawsky, Liming Zhou, Somnath Baidya Roy, Geng Xia, Mathias Vuille, Ronald A. Harris

**Affiliations:** 1 Department of Atmospheric and Environmental Sciences, SUNY at Albany, Albany, NY 12222, USA; E-Mails: lslawsky@albany.edu (L.M.S.); gxia@albany.edu (G.X.); mvuille@albany.edu (M.V.); raharris@albany.edu (R.A.H.); 2 Centre for Atmospheric Sciences, Indian Institute of Technology Delhi, Hauz Khas, New Delhi 110016, India; E-Mail: drsbr@iitd.ac.in

**Keywords:** wind farm impact, atmospheric boundary layer, land surface temperature

## Abstract

This paper assesses impacts of three wind farms in northern Illinois using land surface temperature (LST) data from the Moderate Resolution Imaging Spectroradiometer (MODIS) instruments onboard the Terra and Aqua satellites for the period 2003–2013. Changes in LST between two periods (before and after construction of the wind turbines) and between wind farm pixels and nearby non-wind-farm pixels are quantified. An areal mean increase in LST by 0.18–0.39 °C is observed at nighttime over the wind farms, with the geographic distribution of this warming effect generally spatially coupled with the layout of the wind turbines (referred to as the spatial coupling), while there is no apparent impact on daytime LST. The nighttime LST warming effect varies with seasons, with the strongest warming in winter months of December-February, and the tightest spatial coupling in summer months of June-August. Analysis of seasonal variations in wind speed and direction from weather balloon sounding data and Automated Surface Observing System hourly observations from nearby stations suggest stronger winds correspond to seasons with greater warming and larger downwind impacts. The early morning soundings in Illinois are representative of the nighttime boundary layer and exhibit strong temperature inversions across all seasons. The strong and relatively shallow inversion in summer leaves warm air readily available to be mixed down and spatially well coupled with the turbine. Although the warming effect is strongest in winter, the spatial coupling is more erratic and spread out than in summer. These results suggest that the observed warming signal at nighttime is likely due to the net downward transport of heat from warmer air aloft to the surface, caused by the turbulent mixing in the wakes of the spinning turbine rotor blades.

## 1. Introduction

In 2008 the U.S. Department of Energy outlined an initiative to increase the contribution of wind energy to the US electricity supply to 20% by the year 2030 [1]. The U.S. wind industry had 61,108 megawatts (MW) of installed capacity by the end of 2013, with over 12,000 MW under construction [2]. This would provide enough energy to power 3.5 million American homes. The total installed wind capacity in 2013 is well over seven times the total of 8031 MW a decade earlier in 2003 [2]. Given the current installed capacity and projected increase in wind turbine installations across the globe, assessing potential impacts of wind farms on our environment is of great societal and economic importance.

While converting wind kinetic energy into electricity, spinning turbine blades inevitably create turbulence, modifying surface-atmosphere exchanges of energy, momentum, and moisture, and thus altering near-surface boundary layer profiles and processes [3,4,5]. Most modeling studies indicate that the presence of large groups of wind turbines is expected to have noticeable climatic impacts from local to regional scales, and likely at global scales as well [6,7,8,9,10,11,12]. However, recent simulations project that climatic effects for planned and existing European wind farms remain very small compared to natural climate variability [13]. Such discrepancies result at least partially from uncertainties and deficiencies in model parameterizations as these simulations are rarely validated against observations [14,15]. On the other hand, the effect of wind farms on meteorology is essentially an atmospheric boundary layer (ABL) problem. The detailed physical processes of ABL-turbine interactions remain mostly unknown because of the complexity and heterogeneity of turbulence over different regions.

Though observed meteorological information in wind farms is not readily available to the public, there have been a few field campaigns to assess various impacts and benefits of wind turbines. Ribeiro *et al.* [16] examined the use of wind machine fans for frost prevention over apple orchards, finding them to be up to 60% effective. Baidya Roy and Traiteur [3] observed a warming effect at night and a cooling effect during the day on near-surface air temperatures after analyzing ~1.5 months of observations from a wind farm in California. Rajewski *et al.* [4] showed an apparent increase in air temperature at 9 m height in direct wakes during nighttime and a slight cooling during the day in central Iowa by deploying four flux stations in a wind farm surrounded by cornfields during the summer months in both 2010 and 2011. Smith *et al.* [5] used observations of a large wind farm in the Midwest for ~two weeks in May 2012 and concluded that wind farms co-located with crops have little impact on the vertical temperature structure during the day, but do significantly decrease the vertical gradients of potential temperature, largely by increasing the temperature at 2 m. As the field campaigns are short in time and can only provide measurements at few sites, more observational analyses are needed before extrapolating the results and formulating broad conclusions about wind farm impacts in other regions, particularly over the Midwest and Great Plains, where the wind farm growth rate is among the highest in the United States. 

Since field observations are limited, the use of satellite data is of increasing importance to assess wind farm impacts on larger scales and over longer periods. Zhou *et al.* [17,18,19] examined land surface temperature (LST) changes over wind farms in western Texas using data from the Moderate Resolution Imaging Spectroradiometer (MODIS) instruments onboard the Terra and Aqua satellites. Zhou *et al.* [17] found a nighttime warming effect directly under the turbines and no evidence of daytime impact over the study region from 2003–2011. Zhou *et al.* [18] examined the diurnal and seasonal variations of the warming effect under different quality control of MODIS data. Zhou *et al.* [19] used other approaches to provide more evidence of this warming effect. Their results also indicated that the warming effect is not an artifact of varied surface elevation as most wind turbines were built over high mountain ridges. Walsh-Thomas *et al.* [20] showed similar and complementary results over the San Gorgonio Pass Wind Farm from 1984 to 2011 using higher resolution Landsat data, with a warming trend that is consistently observed downwind of the wind farm. Harris *et al.* [21] analyzed five wind farms in Iowa using MODIS LST data and provided further observational evidence of the wind farm–induced warming effect at nighttime. 

Evaluating different wind farms could shed more light on the spatiotemporal variability of wind farm impacts under different atmospheric, boundary layer and land surface conditions and thus improve our understanding of how wind farms and ABL interact differently over different regions. Furthermore, microclimatic changes over wind turbines such as temperature and humidity changes near the surface could impact underlying vegetation. This is an important issue in the Midwest and the Great Plains where wind farms are often constructed over operating farmlands. Knowledge of these changes would be useful in the development of climate resilient crops and longer lasting turbines.

Remote sensing based studies [17,18,19,20,21] can effectively detect and quantify the wind farm impacts with spatial details from various perspectives (e.g., data quality, topography, land cover/use change), while the physical mechanisms responsible for the wind farm-induced LST changes are mostly speculative because meteorological data were not analyzed. To overcome this limitation, the present study builds upon the research of Zhou *et al.* [17,18] over a region in northern Illinois, which has more variable weather systems than Texas, especially in winter, and very distinct land surface properties, to explore likely physical mechanisms that determine the magnitude and variability of wind farm-induced temperature changes seasonally and diurnally using meteorological data. Large-scale wind power development is a relatively recent phenomenon in Illinois with many wind farms coming online in or around 2003. Hence, Illinois is a good study region to pair with the MODIS Terra and Aqua data that both have full year data starting in 2003. Besides analyzing remote sensed data as done in previous studies [17,18,19,20,21], the roles of atmospheric stability, wind speed and direction in determining the wind farm impacts are primarily examined.

## 2. Data and Methodology

### 2.1. Study Region

**Figure 1:**
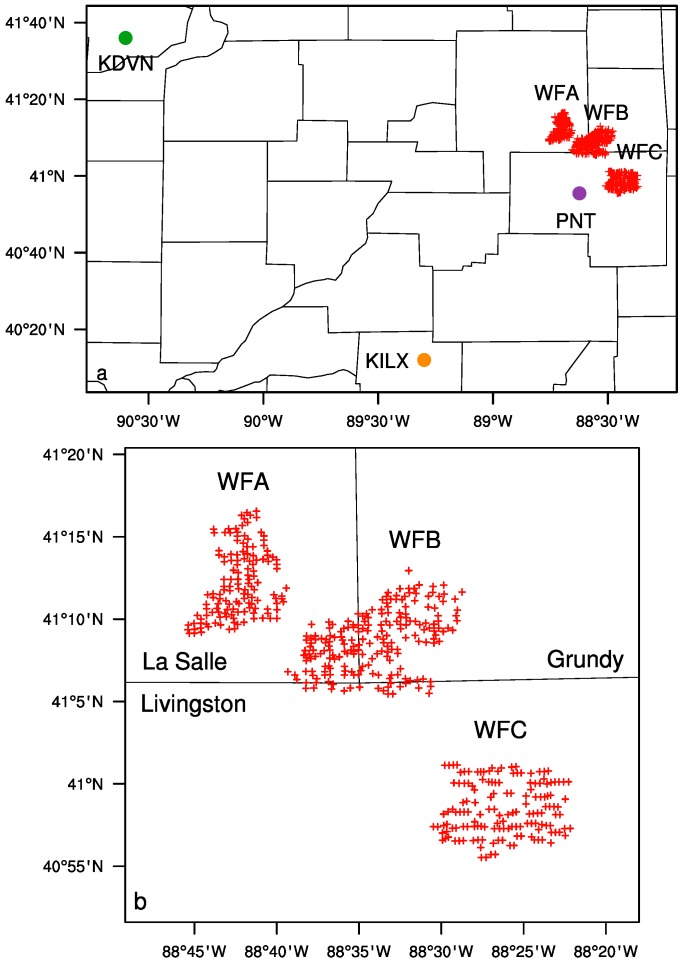

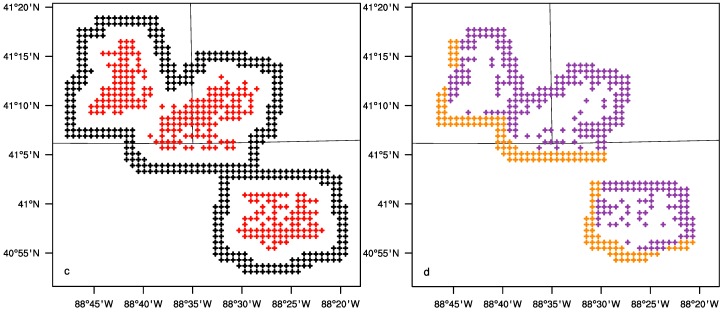
Geographic location of three wind farms labeled WFA, WFB, and WFC, built between 2007 and 2010, together with (**a**) two sounding station sites (KILX and KDVN) and one Automated Surface Observing System (ASOS) station site (PNT) and (**b**) individual wind turbines over the study region (40.86°N–41.34°N, 88.82°W–88.30°W). Plus symbols represent individual wind turbines; (**c**) Definition of wind farm pixels (WFPs, 270 pixels in red) and nearby non-wind farm pixels (NNWFPs, 400 pixels in black) at 0.01° resolution. WFPs contain at least one wind turbine; (**d**) Definition of upwind wind farm pixels (UWFPs, 141 pixels in orange) and downwind wind farm pixels (DWFPs, 300 pixels in blue) at 0.01° resolution. DWFPs and UWFPs are located between WFPs and NNWFPs.

Illinois had the fourth most installed wind power capacity in the United States by the end of 2013 [22]. The study region chosen here (Figure 1) is in the northern part of Illinois (40.86° N–41.34° N, 88.82° W–88.30° W), and lies between La Salle, Livingston, and Grundy counties and encompasses three different wind farms, referred to as WFA, WFB, and WFC. The height of wind turbines, defined as hub-height, is measured by the distance from the turbine platform to the rotor, not including the length of the rotor blades. WFA is located in La Salle County and has 140 General Electric (GE) Company turbines standing at 80 m hub-height with an electrical power output capacity of 1.5 MW each. The construction began in 2007 and it came online (the turbines start spinning and producing electricity) during 2008 and 2009. WFB is located at the boundary of La Salle County and Grundy County, to the north of Livingston County. It consists of 200 1.5 MW GE turbines of 80 m hub-height, coming online in 2009 and 2010. Both WFA and WFB turbines have rotor diameters of about 80 m. WFC is in Livingston County and consists of 150 Gamesa G-87 2.0 MW turbines with 67 m hub-height and 87 m rotor diameter. The construction began in 2009 and WFC came online in 2010.

The geographic location (latitude and longitude) of individual wind turbines within the study region (Figure 1) is identified using the off-airport database of Obstruction Evaluation/Airport Airspace Analysis (OE/AAA) at the Federal Aviation Administration (FAA) as done in Zhou *et al.* [17,18]. 

Initial wind turbine existence is confirmed by built dates from the FAA records for the period 2003–2013 as this database encompasses any development in the study region in Illinois. The Google Earth image history database is then used to confirm the existence and location of each turbine. Also public records and wind farm information on each wind farm validate the total number of turbines [23].

### 2.2. Data

#### 2.2.1. Remotely Sensed Data

This study utilizes MODIS LST and land cover products derived from instruments onboard the NASA’s Terra and Aqua satellites. Terra and Aqua were launched in 1999 and 2002, respectively. Because a full year of data for both Terra and Aqua do not exist for the years 2000–2002, the study period used here begins in 2003. 

The MODIS 8-day LST data at 1 km resolution used are the same as described in Zhou *et al.* [17,18], ranging from 2003 through 2013. The 8-day LST product is the average from 2 to 8 days of daily products to reduce the daily data gap due to the presence of clouds [24]. Each satellite provides two measurements per day and so there are four measurements in total, approximately at local solar times of ~10:30 a.m. and ~22:30 p.m. for Terra (MOD11A2) and ~13:30 p.m. and ~1:30 a.m. for Aqua (MYD11A2). 

Land cover information is obtained from the MODIS land cover map (MCD12Q1) at 500 m spatial resolution. It has five different classification schemes that describe land cover properties [25]. For this study, the International Geosphere Biosphere Programme (IGBP) scheme, which identifies 17 land cover classes, is chosen. There are 11 natural vegetation classes, three non-vegetated classes, and three developed and mosaiced land classes. The land cover map is derived from the combined observations from Terra and Aqua. Because it is composited annually, the first year of the study period (2003) is compared to the last year of data (2012) to detect land cover/use change over the study region. Note that the data in 2013 is not available.

#### 2.2.2. Topography Data

**Figure 2:**
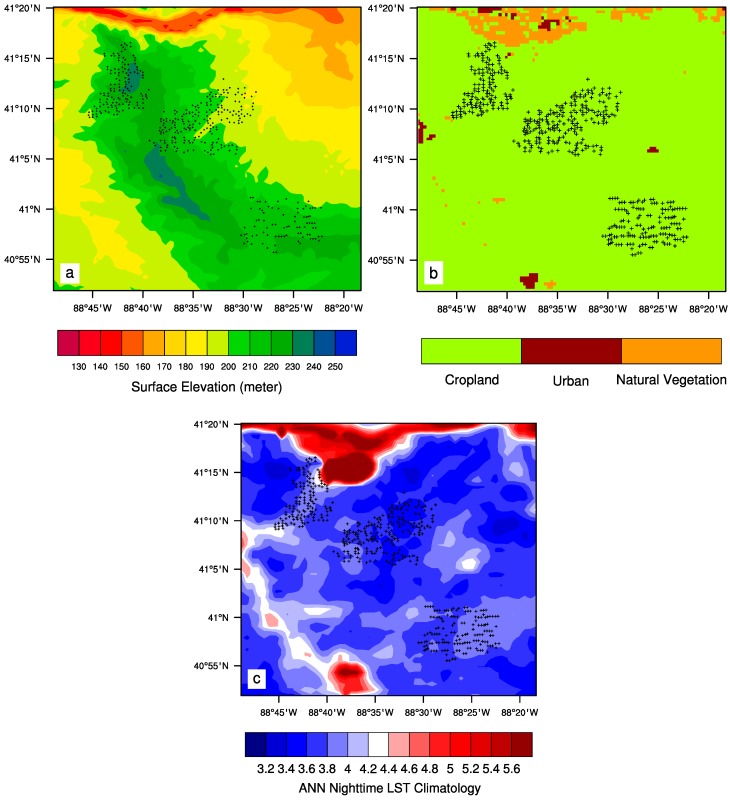
(**a**) Surface topography (m) at ~1 km spatial resolution for the study region; (**b**) Land cover and land use map in 2003 over the study region at 0.005° spatial resolution; (**c**) Spatial pattern of MODIS ANN nighttime LST (°C) climatology at 0.01° spatial resolution over the study region for the period 2003–2013. Plus symbols represent individual wind turbines.

The United States Geological Survey (USGS) provides global 30 arc-second elevation data (GTOPO30) at approximately 1 km spatial resolution. This digital elevation data is divided into tiles, and the West 100 and North 90 tile is used to include the study region (Figure 2a).

#### 2.2.3. Meteorological Data

Surface wind data at 10 m above the ground for the period 2003–2013 are obtained from an Automated Surface Observing System (ASOS) station, located in Pontiac, Illinois (PNT), which lies 32 km south of the wind farms (Figure 1a). The ASOS data is retrieved from the Iowa Environmental Mesonet (IEM) and provide measurements at 20-minute intervals. Here we use ASOS to provide wind statistics at a location closer to the wind farms and at a higher temporal resolution covering the Terra and Aqua measurement times than sounding data. Because the majority of turbines in the study region stand at an 80 m hub-height, and the measurements are taken only at 10 m, there will be an increase in wind speed at the higher hub-height due to lessened impacts from near surface roughness.

Note that vertical profiles of wind and temperature are useful to study ABL processes at the turbine hub-height but sounding data from weather stations can only provide measurements twice daily (00 and 12 GMT). Nevertheless, two weather stations with sounding data near the wind farms are used and compared for quality assurance. Lincoln, Illinois (04833, KILX) lies about 145 km south of the wind farms; Davenport, Iowa (94982, KDVN) lies about 200 km west (Figure 1a). The atmospheric sounding data used in this study are retrieved from the Stratosphere-troposphere Processes And their Role in Climate (SPARC) website. This high resolution data consists of measurements taken every second during the ascent of the balloon for a time period ranging from 2008 through 2011. Though this does not cover the entirety of the study period, it may be sufficient to collect a climatological signature of the atmospheric profiles during the times when the wind turbines were operating. The station surface elevation is 179 m for KILX and 230 m for KDVN. As the average elevation of the wind turbines is 213 m, we choose the sounding data around 293 m above sea level to represents the 80 m hub-height measurements. We analyzed the seasonal variations of wind profiles, which show similar features as the ASOS data but with a larger magnitude. We also analyzed the temperature profiles to examine the seasonal variations of the strength and depth of the temperature inversion layer. 

### 2.3. Data Processing

For each year there are 184 LST images consisting of 46 8-day composites and four measurements per composite from both the Terra and Aqua satellites. The MODIS 8-day LST data is combined into monthly means and is used to create monthly anomalies by subtracting the LST climatology at every pixel. The data are then further combined into seasonal mean and anomalies in DJF (Dec-Jan-Feb), MAM (Mar-Apr-May), JJA (Jun-Jul-Aug), and SON (Sep-Oct-Nov). Annual (ANN) means and anomalies are also created. There are eleven years of LST data for the period 2003–2013. For each season and ANN in each year, there are four images of LST corresponding to the four acquisition times of the MODIS data.

The wind farm-induced LST changes are a low-frequency signal while the background LST has a strong diurnal, seasonal and interannual variability, which is a high-frequency signal. In particular, the interannual variability is strong, especially in DJF. Hence, the two MODIS LST data for daytime (~10:30 a.m. and ~13:30 p.m.) and nighttime (~22:30 p.m. and ~1:30 a.m.) are combined into one daytime and one nighttime image in each season and in ANN for every year as done in Zhou *et al.* [17]. This averaging should reduce data noise while minimizing high-frequency variations from daily weather events and changes in solar heating [26] and thus make the low-frequency signal easy to be detected [17,18,19,21].

The MODIS LST data on a sinusoidal projection are re-projected into pixels with 0.01° resolution (~1.1 km), which is slightly coarser than the original 1 km data in order to avoid spatial gaps. The study region consists of 2496 pixels (52 columns, 48 rows) covering the total of 490 wind turbines (Figure 1). Similarly, the MODIS land cover map is re-projected from the 500 m spatial resolution of the original data set to a 0.005° spatial resolution plot. The ASOS wind speed and direction observations are composited into hourly means by seasons as well. The daily sounding data are composited into seasonal means similarly.

### 2.4. Detection and Attribution Methods

#### 2.4.1. Definition of Four Groups of Pixels

Four groups of pixels at 0.01° spatial resolution (Figure 1c,d) are defined to quantify the LST changes and their association with the wind turbines. We define those pixels containing at least one wind turbine as wind farm pixels (WFPs), and those non-wind-farm pixels surrounding each wind farm as nearby non-wind-farm pixels (NNWFPs). NNWFPs are selected at a distance of 2 pixels away from WFPs. Turbulence decreases farther downwind from the rotor blades [7], and a distance of ten rotor diameters downstream is enough for the flow to recover from the turbulence caused by the rotor blades [27,28]. For example, the 1.5 MW GE wind turbines have a rotor diameter of ~80 m, meaning 10 rotor diameters away would be 800 m [29]. The distance used here of 2 pixels at the 0.01° resolution, is over 2 km away. Even if wakes were to extend to 15 rotor diameters [30], the distance here is still large enough to minimize most impacts from turbulence downwind of the rotors. In total, there are 270 WFPs and 400 NNWFPs (Figure 1c). The presence of wind turbines will have an effect on their downwind pixels. We further classify the 441 pixels between WFPs and NNWFPs into two groups: upwind wind farm pixels (UWFPs, 141 pixels) and downwind wind farm pixels (DWFPs, 300 pixels) (Figure 1d). As the wind direction is primarily from south and west over the study region (see more discussion in Section 3.3.4), DWFPs contain those pixels in the east and north of WFPs, and UWFPs have those pixels in the west and south of WFPs. 

#### 2.4.2. Spatial Pattern Analysis

The average LST anomalies during the pre-turbine construction period (2003–2006) are subtracted from those during the post-turbine construction period (2010–2013) at the pixel level. As the background LST varies substantially from one year to another, we remove the regional mean LST anomaly (one constant value) from the resulting LST anomaly image to highlight the sub-regional LST spatial variability as detailed in Zhou *et al.* [17,18]. By evaluating the relative LST differences between these two periods, a warming or cooling signal in LST will become apparent across the study region. If the geographic distribution of the LST changes is generally spatially coupled with the layout of the wind turbines (referred to as the spatial coupling hereafter), it suggests potential impacts of the wind farms on LST. 

This spatial coupling can be checked by examining the spatial distribution of LST changes *versus* the layout of wind farms but is also quantified by a simple method introduced here. We rank the LST differences from all of the 2496 pixels within the study region in a decreasing order and pick up the first 5%, 10% and 15% pixels with the largest LST anomalies (the strongest warming signal). We calculate the percentage of these 5%, 10% and 15% pixels falling into each of the four groups of pixels, referred to as the spatial coupling index (SCI). If the LST changes are randomly distributed spatially, the possibility of these pixels falling into the four groups (referred to as the SCI threshold) should be 10.8% (270 pixels divided by 2496 pixels) for WFPs, 16.0% (400 pixels divided by 2496 pixels) for NNWFPs, 5.6% (141 pixels divided by 2496 pixels) for UWFPs, and 12.0% (300 pixels divided by 2496 pixels) for DWFPs. The SCI value can be used to quantify the degree of spatial coupling or downwind effect of the LST changes with the wind farms. 

#### 2.4.3. Temporal Variability Analysis

The areal mean interannual LST differences (WFPs minus NNWFPs) are examined for each season to assess the wind farm impacts on temperature. It is reasonable to assume that WFPs and NNWFPs share similar background meteorological conditions and so their LST differences reflect primarily impacts of local land surface conditions. If the operational wind farms cause a warming effect, the LST differences should exhibit positive values after the wind farms are built. 

We quantify the wind farm impact by calculating the areal mean LST differences (WFPs minus NNWFPs) between the post- and pre- turbine construction periods (2010–2013 minus 2003–2006). Positive values mean warmer temperatures over WFPs relative to NNWFPs, suggesting an impact from the operational wind turbines. It is important to note that prior to the existence of the wind turbines, WFPs and NNWFPs don’t have identical temperatures due to land surface heterogeneity. The underlying vegetation, though similar, is not expected to have exactly the same thermal effects due to variations in vegetation amount/cover. That is why we need to calculate the areal mean LST difference between the post- and pre- turbine construction period to remove the LST difference between WFPs and NNWFPs even before the turbines are built.

## 3. Results and Discussion

### 3.1. Spatial Patterns of Wind Farm Impacts on LST

Land surface properties largely determine the climatology of LST. The MODIS land cover map (Figure 2b) shows that most of the study area under the turbines is uniformly used for crops (green). The land cover types include grassland, shrubs, forests, and cropland. There are also small urban areas (in maroon) to the north and south of the wind farms, and natural vegetation (in orange) to the north along the river, which can be identified easier in the local topography map (Figure 2a). Figure 2c displays the climatology of ANN nighttime LST for the period 2003–2013. Evidently, the spatial variations in LST are associated with the surface topography (Figure 2a) and land cover map (Figure 2b). Several large towns show an urban heat island effect. The river and its nearby vegetation are warmer than the cropland at night because the former has higher heat capacity than the latter. Effects due to surface heterogeneity like these are minimized by using the LST anomalies (deviations from climatological means) at pixel level, as land cover and topography do not change much from one year to the next.

**Figure 3:**
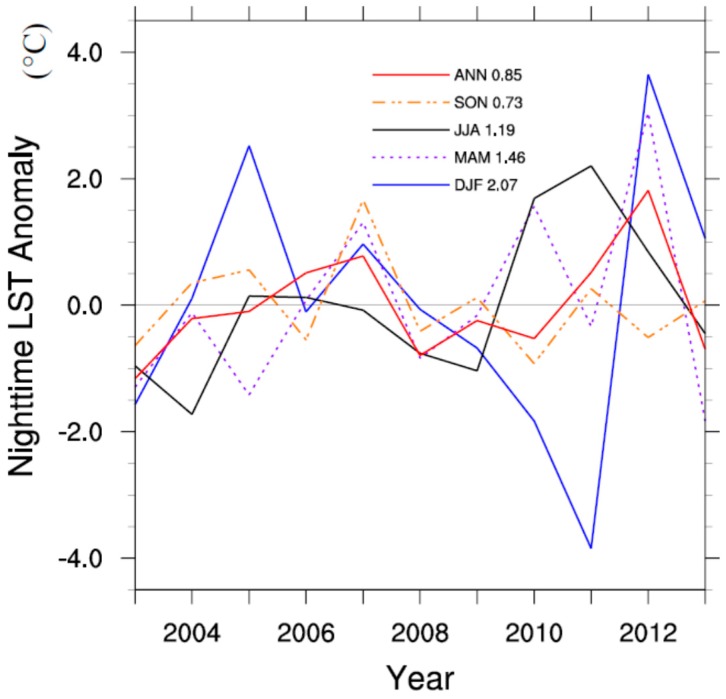
Regional mean interannual MODIS nighttime LST anomalies (°C) over the study region for the four seasons (DJF, MAM, JJA, and SON) and annual mean (ANN). Standard deviations of LST anomalies are shown.

The LST shows strong interannual variability over the study region. Figure 3 illustrates the regional mean LST anomalies at nighttime and their standard deviations (STDs) from 2003 to 2013. DJF LST changes substantially due to variable winter weather systems each year. MAM LST also displays much variability from year to year. Above average LSTs in these seasons greatly impact the ANN LST in 2007 and 2012. A strong negative LST anomaly in DJF in 2011 of nearly 4.0 °C below average reduced the annual LST, even though this year was 2.0 °C warmer than average in JJA. The STD is largest in DJF (2.07 °C), followed by MAM (1.46 °C), JJA (1.19 °C), and SON (0.73 °C). The high-frequency LST variability will mask to some extent the wind farm induced low-frequency LST signal [18,19], particularly in the winter months (see more discussion later). 

**Figure 4:**
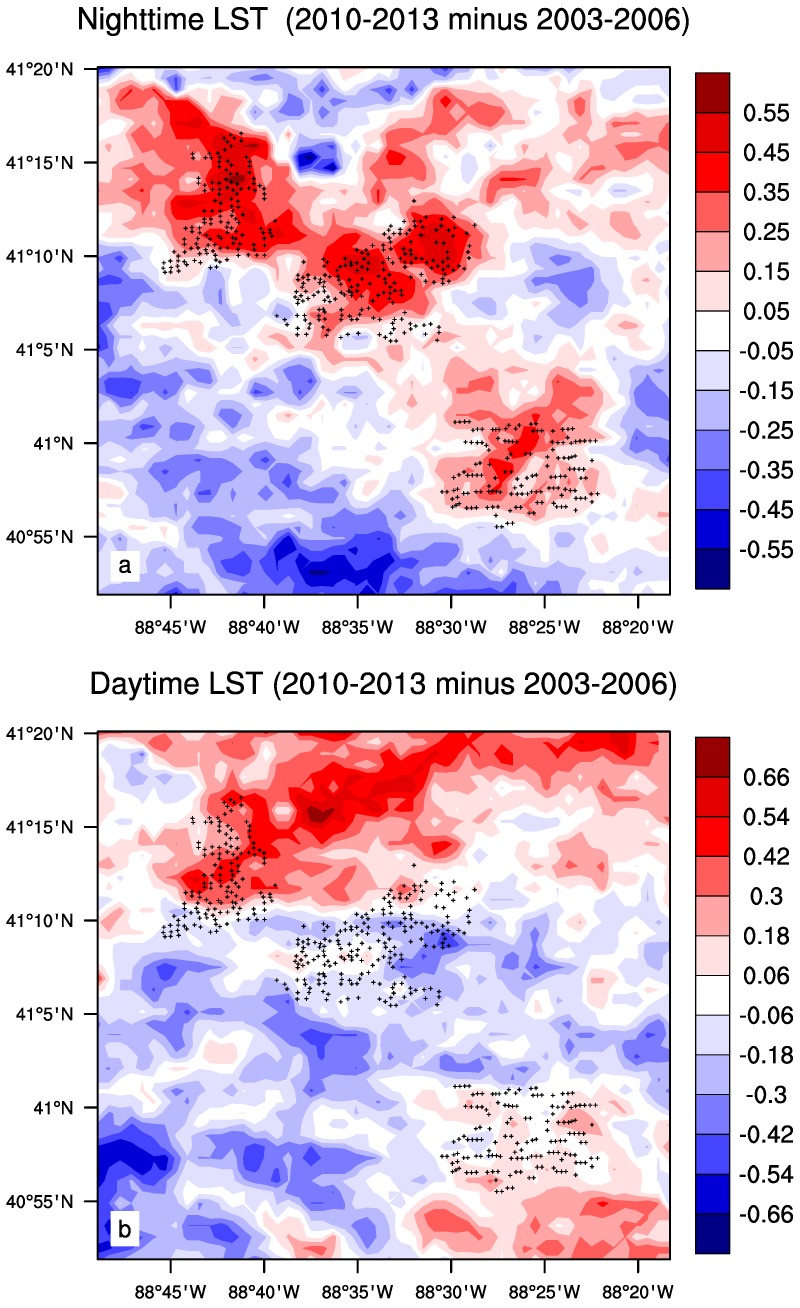
Annual mean MODIS LST differences (°C) between two periods (2010–2013 minus 2003–2006 averages) at (**a**) nighttime and (**b**) daytime. Plus symbols represent pixels with at least one wind turbine.

Figure 4 shows the ANN LST differences between the pre-turbine construction period (2003–2006) and the post-turbine construction period (2010–2013) at the pixel level. Consistent with the previous studies [17,18], the positive LST anomalies are spatially coupled with the wind turbines at nighttime, but the daytime LST does not display any spatial coupling with the wind turbines. Therefore, daytime results are excluded from this study for the sake of brevity. 

**Figure 5:**
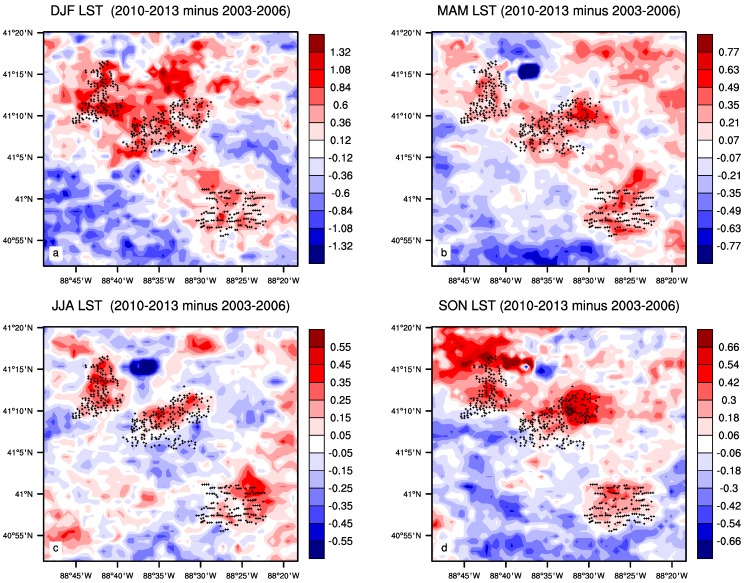
Seasonal mean MODIS nighttime LST differences (°C) between the pre- and post- turbine construction period (2010–2013 minus 2003–2006 averages) in (**a**) DJF; (**b**) MAM; (**c**) JJA and (**d**) SON. Plus symbols represent pixels with at least one wind turbine.

The nighttime LST difference plots between the pre- and post- turbine construction period for each season are shown in Figure 5. The spatial coupling of warming with the wind turbines is evident in every season, and this coupling is the tightest in JJA, followed by SON and MAM, and the weakest in DJF, which is consistent with the quantitative analysis using the SCI index described below. In general, the shift of the warming toward the northeast corresponds to climatological wind directions from the west, southwest and south, and the magnitude of warming corresponds to the strength of wind speed at hub-height (see more discussion later). The strength of the spatial coupling is also related to the magnitude of inter-annual variability in LST (Figure 3) associated with variable weather events: larger variability causes the widespread warming seen in MAM and DJF, while less variability in SON and JJA generally mimic the overall annual variations, which are consistent with Zhou *et al.* [18]. Consequently, JJA, SON, and ANN have the tightest spatial coupling of nighttime warming with the wind turbines (Figure 4 and Figure 5).

We quantify this spatial coupling by comparing the SCI values of the top 5%, 10% and 15% pixels with the strongest warming anomalies falling into WFPs compared with the corresponding SCI threshold (10.8%) (Table 1). It should be noted that the higher the SCI, the stronger the spatial coupling. Evidently, SCI ranges seasonally from 25.7% to 40.3% for WFPs, significantly higher than the 10.8% SCI threshold, pointing to the wind farms as cause of this warming effect. Generally JJA has the highest SCI value, followed by SON and MAM, while DJF has the smallest SCI value. Compared to the seasonal SCI statistics, ANN has the highest SCI values (36.1%–54.8%) and thus the tightest spatial coupling because it has the least high-frequency LST variations that likely mask the detection of the wind farm-induced low-frequency warming signal. 

**Table 1:** Seasonal variations in the percentage of the top 5%, 10% and 15% pixels with the strongest warming anomalies falling into each group of pixels (referred to as spatial coupling index (SCI))

Number of Pixels (% of Total Pixels)	Season	WFPs	NNWFPs	UWFPs	DWFPs
Random	SCI threshold	10.8	16.0	5.6	12.0
124 (5%)	DJF	**33.1**	**18.5**	3.2	**29.0**
	MAM	**29.0**	6.5	0.0	**23.4**
	JJA	**40.3**	8.1	0.8	**20.2**
	SON	**31.5**	**20.2**	4.0	**23.4**
	ANN	**54.8**	7.3	0.0	**29.8**
249 (10%)	DJF	**27.7**	15.7	5.2	**29.7**
	MAM	**29.3**	9.2	1.6	**19.3**
	JJA	**33.7**	7.6	0.4	**16.1**
	SON	**30.5**	14.1	3.6	**23.3**
	ANN	**42.2**	8.8	2.0	**30.1**
374 (15%)	DJF	**27.0**	14.4	**6.7**	**28.1**
	MAM	**25.7**	9.9	2.7	**19.0**
	JJA	**29.9**	7.0	0.3	**13.4**
	SON	**28.6**	13.1	2.4	**23.5**
	ANN	**36.1**	11.2	2.1	**28.9**

WFPs, NNWFPs, DWFPs, and UWFPs are defined in the main text and shown in Figure 1. Values in bold (rows 2–16) are larger than those that would be generated randomly (the first row).

### 3.2. Temporal Variability of Wind Farm Impacts on LST

**Figure 6:**
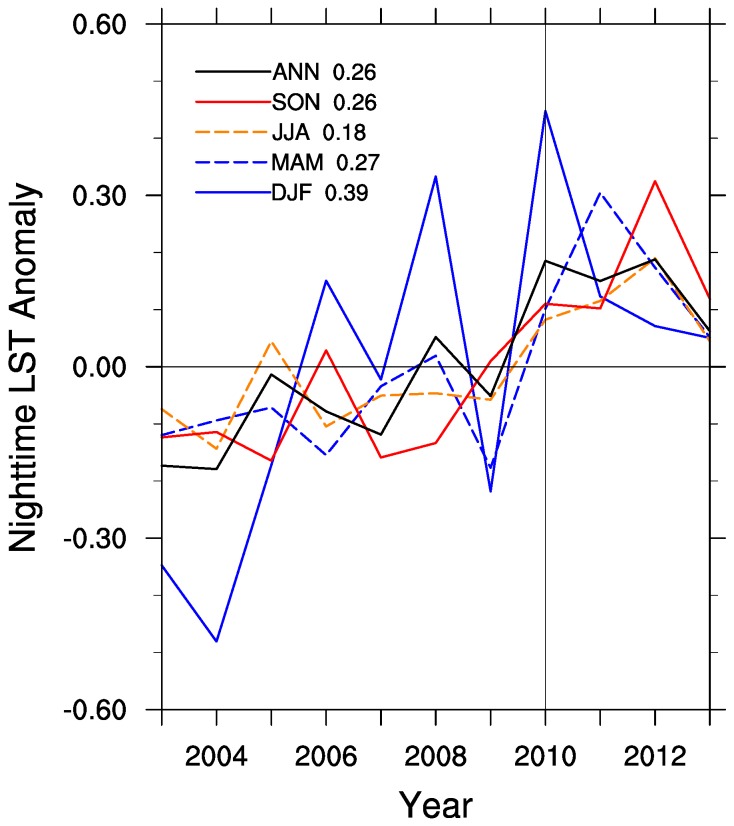
Areal mean MODIS nighttime LST anomalies (°C) between WFPs and NNWFPs (WFPs minus NNWFPs) for the period 2003–2013. The vertical reference line at the year 2010 indicates wind turbine construction completion for all three wind farms. The areal mean LST anomaly differences between the post- and pre-turbine construction periods (2010–2013 minus 2003–2006) are shown.

Figure 6 shows the areal mean seasonal and ANN time series of LST differences (WFPs minus NNWFPs) from 2003 to 2013. Despite a strong interannual variability, there is clearly a jump toward positive values across all seasons after 2010, when all the turbines came online, suggesting that the construction of wind turbines warm WFPs relative to NNWFPs. We can quantify the magnitude of the warming effect by subtracting the LST anomalies between the pre- and post-turbine construction period (2010–2013 minus 2003–2006). The warming rate is found to be the largest in DJF (0.39 °C), followed by MAM (0.27 °C) and SON (0.26 °C), and smallest in JJA (0.18 °C). The annual mean LST exhibits a warming effect of 0.26 °C.

### 3.3. Possible Factors Determining Wind Farm Impacts

#### 3.3.1. Impacts of Topography and Land Use Change

Wind farm impacts on LST are small in magnitude, compared to the background year to year variations in LST, and can be modified by changes in topography and land cover/use. The local surface elevation (Figure 2a) varies only within 30 m across the wind farms. If there was a topographical impact on the nighttime warming signal, the LST differences between the pre- and post-turbine construction period would diminish with height around the wind turbines [19]. This is not seen during any season (Figure 4 and Figure 5) and particularly the warming signal does not show any spatial connections to the surface elevation. This suggests that elevation variations are small enough to have a negligible influence on LST.

**Figure 7:**
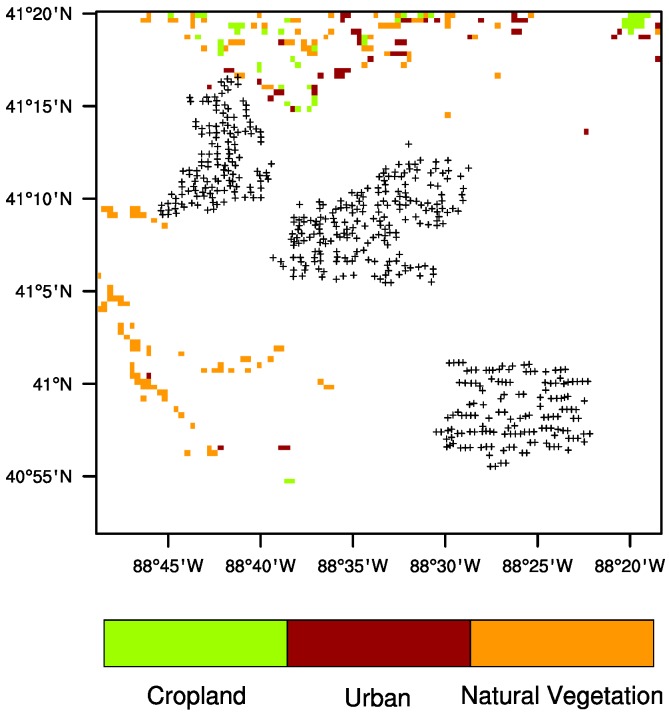
Land cover and land use change map from 2003 to 2012 over the study region at 0.005° spatial resolution. Plus symbols represent pixels with at least one wind turbine. White represents no change, orange a change to natural vegetation, maroon a change to urban, green a change to crop.

The MODIS land cover change map from 2003 to 2012 (Figure 7) indicates no land cover/use change occurred directly under the wind farms. Few surrounding areas have changed toward more natural vegetation to the southwest and some urban sprawl occurred to the north. These changes should result in some small changes in LST, particularly at daytime. However, these pixels will not affect our quantification of wind farm impacts as they are very limited in size and located away from our studied groups of pixels. 

#### 3.3.2. Impacts of Temperature Inversion

During the day, the ABL is well mixed as the sun heats the ground, allowing warmer air parcels to convectively rise and cause turbulence which mixes the atmosphere [31]. Once the sun sets, the ground cools radiatively and there are no more parcels to rise and mix the atmosphere, thus turbulence is reduced near the surface and a stable boundary layer (SBL) forms through the night. In the stratified nocturnal SBL, lighter warm air overlies the denser surface cool air, creating a temperature inversion [32]. The spinning rotor blades mix the atmosphere, and bring the warmer fast-moving air from aloft down to the surface [3]. Therefore, the warming LST signal at nighttime, which is spatially coupled with the wind turbines, is likely due to downward transport of warmer air aloft to the surface by the turbulence caused by the spinning rotor blades as discussed next.

**Figure 8:**
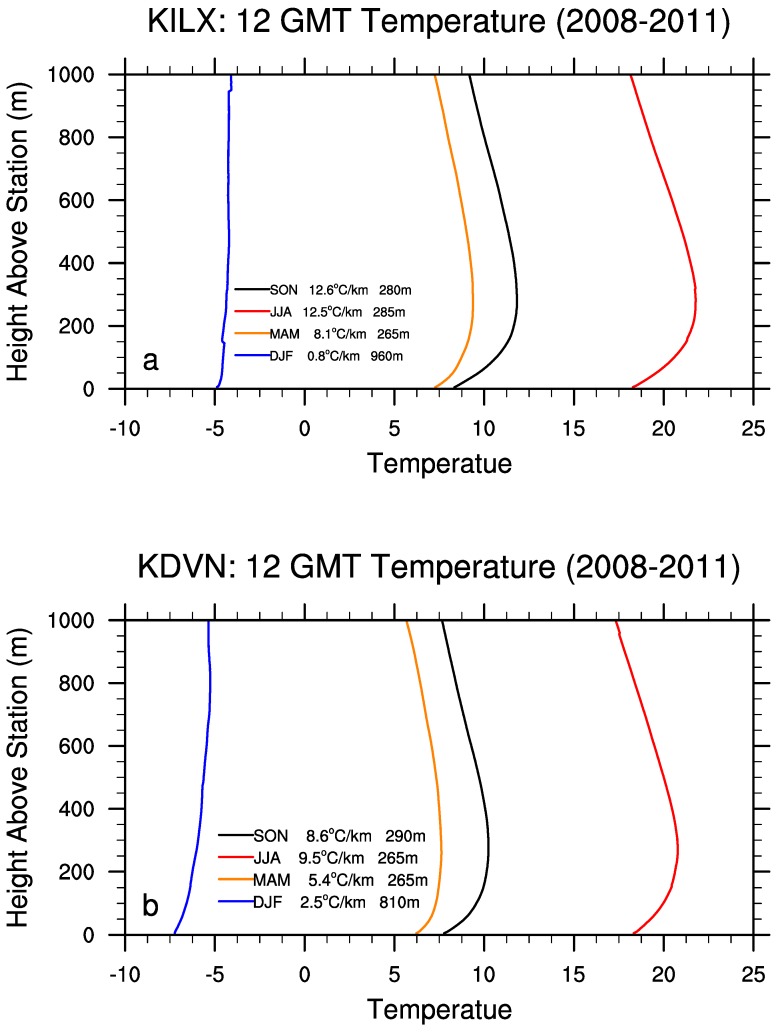
Seasonal mean 12:00 GMT temperature vertical profiles (°C) as a function of height in meters for (**a**) Lincoln, IL (KILX) and (**b**) Davenport, IA (KDVN). The temperature gradient (°C/km) between the station surface and ~80 m hub-height (~293 m above sea level) and the depth of near surface boundary layer (m), where the temperature maximizes, are shown. The station surface elevation is 179 m for KILX and 230 m for KDVN.

Vertical temperature profiles from the two sounding locations (Figure 8) reflect this temperature inversion with increasingly warmer air aloft from the surface up to about 900 m. We use the early morning 12:00 GMT (6:00/7:00 a.m. local solar time) sounding data, which is assumed to be representative of the nighttime SBL. This assumption is truer in the winter than in the summer. However, the temperature profiles (Figure 8) show a stable condition during early morning hours in all seasons, indicating that similar stability conditions also prevailed in the night before. A departure from this pattern may occur if the air temperature suddenly drops in cases such as if there is sudden influx of cold air mass right around or after sunrise. It is unlikely that such events occur every day or frequently enough to change the seasonal mean stability pattern. Hence, from Figure 8 we can reasonably conclude that the nocturnal environment is stable as well.

At both locations, the warmest air temperatures and strongest inversions occur in JJA and SON with a temperature gradient of 8.6–12.6 °C/km from the surface up to the maximum temperature at 265–290 m. The DJF inversion is smallest in magnitude at both KILX (0.8 °C/km) and KDVN (2.5 °C/km), and extends higher up into the atmosphere with the maximum temperature at 960 m and 810 m, respectively. MAM also shows the temperature inversion profiles, 8.1 °C/km at KILX and 5.4 °C/km at KDVN, respectively, and the inversion layer depth is 265 m. Among the four seasons, JJA and SON have the strongest inversion, followed by MAM, and DJF has the weakest inversion. The lower the inversion layer, the stronger the temperature gradient and the more warm air available to be brought down to the surface by the rotor blades. The strong and relatively shallow inversion in JJA and SON leaves warm air readily available to be mixed down and well spatially coupled with the turbine. Although the warming effect is the strongest in DJF and MAM, the spatial coupling is more erratic and spread out, as wind speeds are faster and strong disturbances occur due to more frequent passing of synoptic-scale weather events during these two seasons.

#### 3.3.3. Impacts of Wind Speed

**Figure 9:**
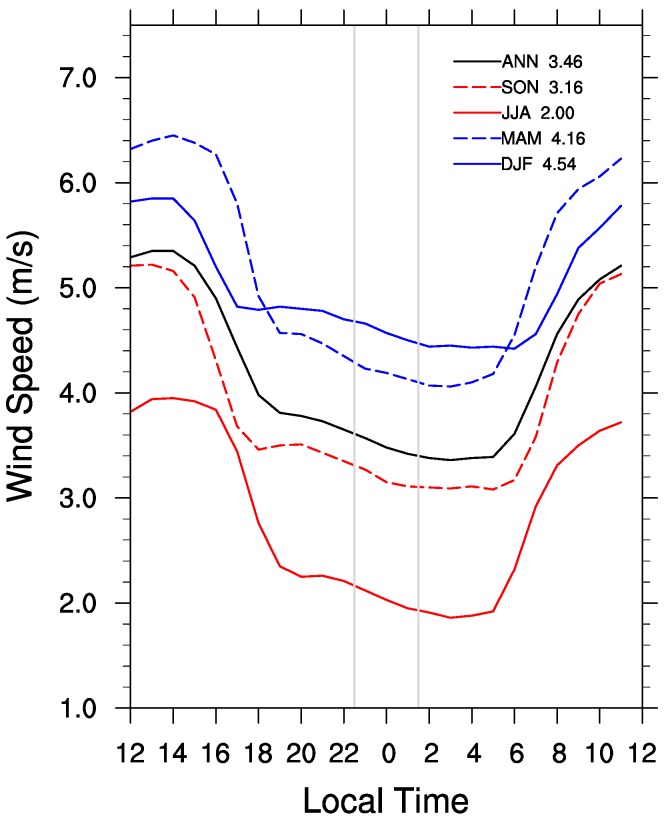
ASOS seasonal mean diurnal (hourly) cycle of wind speed (m/s) at 10 m height for Pontiac, IL (PNT). The two vertical gray lines represent the Terra and Aqua data collection times at ~22:30 p.m. and ~1:30 a.m. The x-axis is time starting at 12 noon on the left, 0 mid-night in the center, and 11 a.m. on the right. Seasonal mean wind speed (m/s) at 10 m averaged between 22:00 p.m. and ~2:00 a.m. is shown.

The diurnal cycle of hourly seasonal composite wind speed (Figure 9) is representative of the turbulence caused by rising thermals in the daytime and calmer surface wind speeds at night in the SBL. Overall slower wind speeds are seen at nighttime with winds ramping up during the day due to solar heating. As the sun heats the ground, rising warm air mixes the atmosphere and faster winds are produced through all levels. At night with no solar heating the atmosphere is able to become stably stratified again as a result of radiative cooling. The wind speed averaged during the MODIS nighttime collection times (Figure 9) is strongest in DJF (4.54 m/s), followed by MAM (4.16 m/s), SON (3.16 m/s) and JJA (2.00 m/s). Overall, the wind speed in JJA is evidently smaller than that in the other three seasons.

The ASOS station in Pontiac, IL (PNT) has a fixed measurement height of 10 m and it can therefore be assumed that the wind speeds at 80 m are faster as they are farther from the surface with less roughness to interfere with the dominant wind flow. Values for wind speed at 80 m hub-height are often extrapolated from wind speed measurements at 10 m above ground level (e.g., from ASOS observations) using the power law [33,34,35]:

(1) $$V\left(z\right)=V_{R}{\left(\frac{z}{z_{R}}\right)}^{\propto }\;$$

where *V(z)* is the wind speed at the desired height *z* of 80 m, *V_R_* is the wind speed from the ASOS station at the reference elevation *z_R_* (10 m), and α is a shear exponent that governs the amount of wind shear between the reference height and the turbine hub height. Traditionally, neutral atmospheric conditions have been associated with α = 1/7, with values higher (lower) than 1/7 indicating stable (unstable) conditions [33,35]. Using the Oklahoma Mesonet data, Newman and Klein [35] found that the power law method performs better than similarity theory methods, particularly under stable conditions, and can be easily applied to wind speed data from different seasons. They showed that α can reach 0.39 under strongly stable atmospheric conditions. It is clear that the stable nocturnal environment over our study region is not neutral and so α could vary between 1/7 and 0.39. Note that Equation (1) is used here to approximate the hub-height wind speeds corresponding to the MODIS LST measurement times. For α = 1/7, Equation (1) gives the hub-height wind speed of 6.11 m/s in DJF, 5.60 m/s in MAM, 4.25 m/s in SON and 2.69 m/s in JJA. For α = 0.39, the corresponding wind speed is 13.75 m/s in DJF, 12.60 m/s in MAM, 9.57 m/s in SON and 6.06 m/s in JJA. It is clear that the former gives lower estimates than the latter. Nevertheless, both cases show that the resulting wind speed at 80 m hub-height is strongest in DJF, followed by MAM, SON and JJA. 

**Figure 10:**
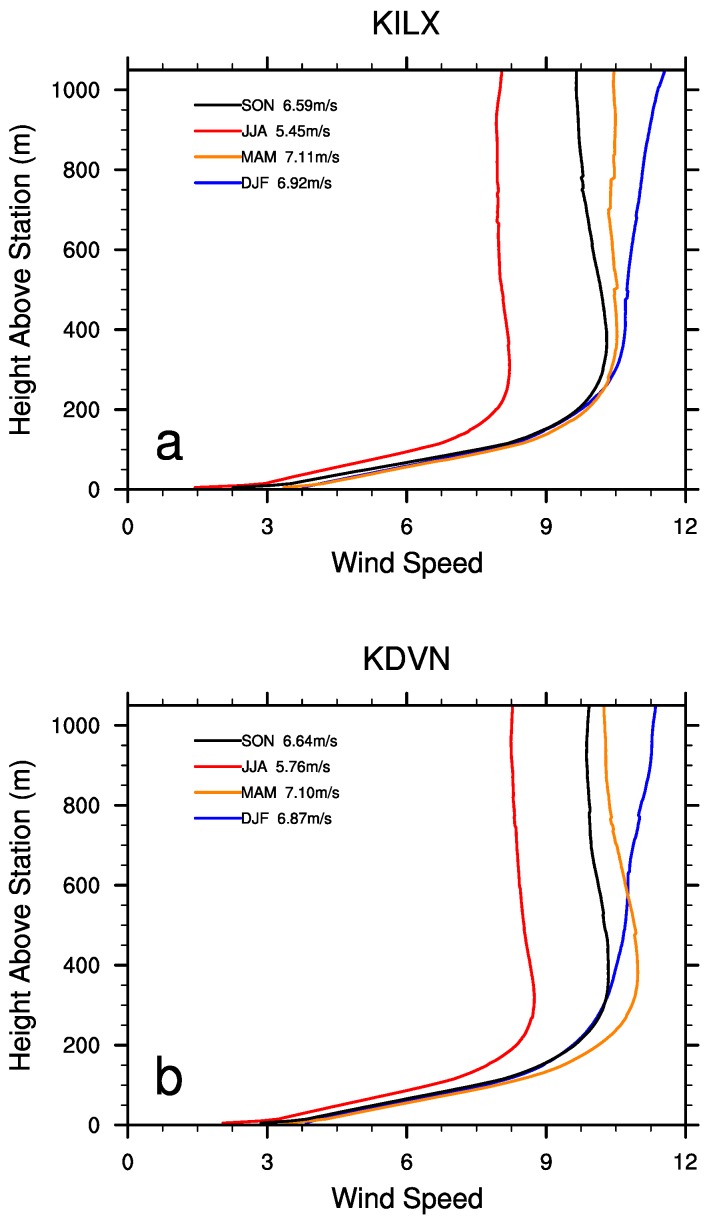
Seasonal mean 12:00 GMT wind speed (m/s) vertical profiles as a function of height in meters for (**a**) KILX and (**b**) KDVN. Seasonal mean wind speed (m/s) at ~80 m hub height (~293 m above sea level) is shown. The station surface elevation is 179 m for KILX and 230 m for KDVN.

Composited vertical profiles of wind speed at 12:00 GMT are plotted for every season from each sounding station from the surface up to about 1000 m to display wind speeds around the rotor blades. Both sounding stations (Figure 10) reflect an increase in wind speed with altitude from the surface, which is expected given boundary layer laws, and show similar wind profiles. Averaged measurements closest to the 80 m hub-height are used to determine seasonal composite wind speeds around the rotor blades (Figure 10). The mean wind speed is strongest in MAM (7.11 m/s at KILX and 7.10 m/s at KDVN), followed by DJF (6.92 m/s at KILX and 6.87 m/s at KDVN), SON (6.59 m/s at KILX and 6.64 m/s at KDVN), and JJA (5.45 m/s at KILX and 5.76 m/s at KDVN). Note that the sounding data at 1200 GMT correspond to local solar time 6:00/7:00 a.m. over Northern Illinois when the wind speed in MAM becomes larger than that in DJF. If interpolated to the MODIS measurement times following the ASOS hourly wind speed diurnal cycle (Figure 9), the hub-height wind speed should be strongest in DJF, followed by MAM, SON and JJA, which are consistent with the ASOS results shown above. Overall, the wind speed is strongest in DJF and weakest in JJA.

It should be noted that the cut-in wind speed, or the speed at which the rotor blades begin to turn and generate electricity, for a GE wind turbine (which comprise the majority of the turbines in the study region) is 3.5 m/s, and the cut-out speed, or the speed at which the turbines are shut down because winds are too strong, is around 20 m/s [29]. The wind speeds at hub-height given above are in the cut-in and cut-out speed range for all seasons except for JJA, indicating on average, the wind turbines are operating across the year except for some days in summer, not only generating electricity, but also mixing the atmosphere, bringing down warm air, and creating turbulence during the night.

It is interesting to see that the nighttime warming effect is strongest in DJF, followed by MAM, SON and DJF (Figure 6), which correspond well to the magnitude of wind speed seasonally (Figure 9a). Also the spatial coupling of the warming rate with the turbines is the tightest in JJA and the weakest in DJF. It may follow that slower wind speeds allow for less dispersion of the warming signal around the turbines, resulting in a better spatial coupling. So the lowest wind speeds in JJA may partially contribute to the best spatial coupling, as the air brought down is less turbulent, leading to a warm LST signal more tightly constrained near the turbines. The stronger wind speeds on average in MAM and DJF, also due in part to more frequent passing of synoptic-scale weather systems, may contribute to the more widespread LST warming signal seen in these two seasons.

#### 3.3.4. Impacts of Wind Direction

Wind direction is also an important factor for considering the downwind impacts. Seasonal shifts in the spatial coupling between nighttime LST warming and wind turbine clusters could be due to changing wind directions. Wind direction changes with height from the top of the surface layer to the top of the ABL but it remains unchanged within the surface layer [31]. As the surface layer is about 10% of the ABL height, wind changes its direction from the surface to 80 m hub-height at night when the ABL is shallow (several hundred meters) but weak during the day when the ABL is deep (1 km or more). Although ASOS can provide wind statistics at a location closer to the wind farms and at a higher temporal resolution covering the satellite measurement times than sounding data, the 10 m wind direction may not be good to estimate the wind direction at the hub-height. Therefore, here we use the sounding data to examine possible impacts of wind direction on the LSTs. 

**Figure 11:**
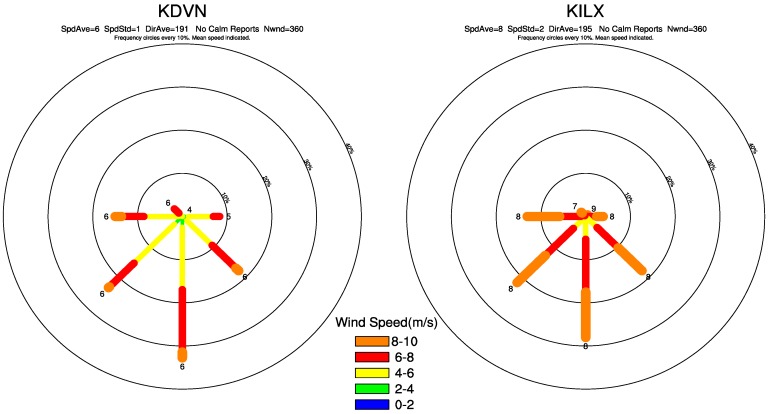
Wind rose for for the sounding stations of KDVN (**left**) and KILX (**right**) from the annual (ANN) sounding composites observed ~80 m hub height (~293 m above sea level) at 12:00 GMT. The station surface elevation is 179 m for KILX and 230 m for KDVN. Wind rose displays the percentage, or frequency of wind directions and the winds from the north are at the top of the rose. The mean wind speed (m/s) is labeled at the end of each direction line.

**Figure 12:**
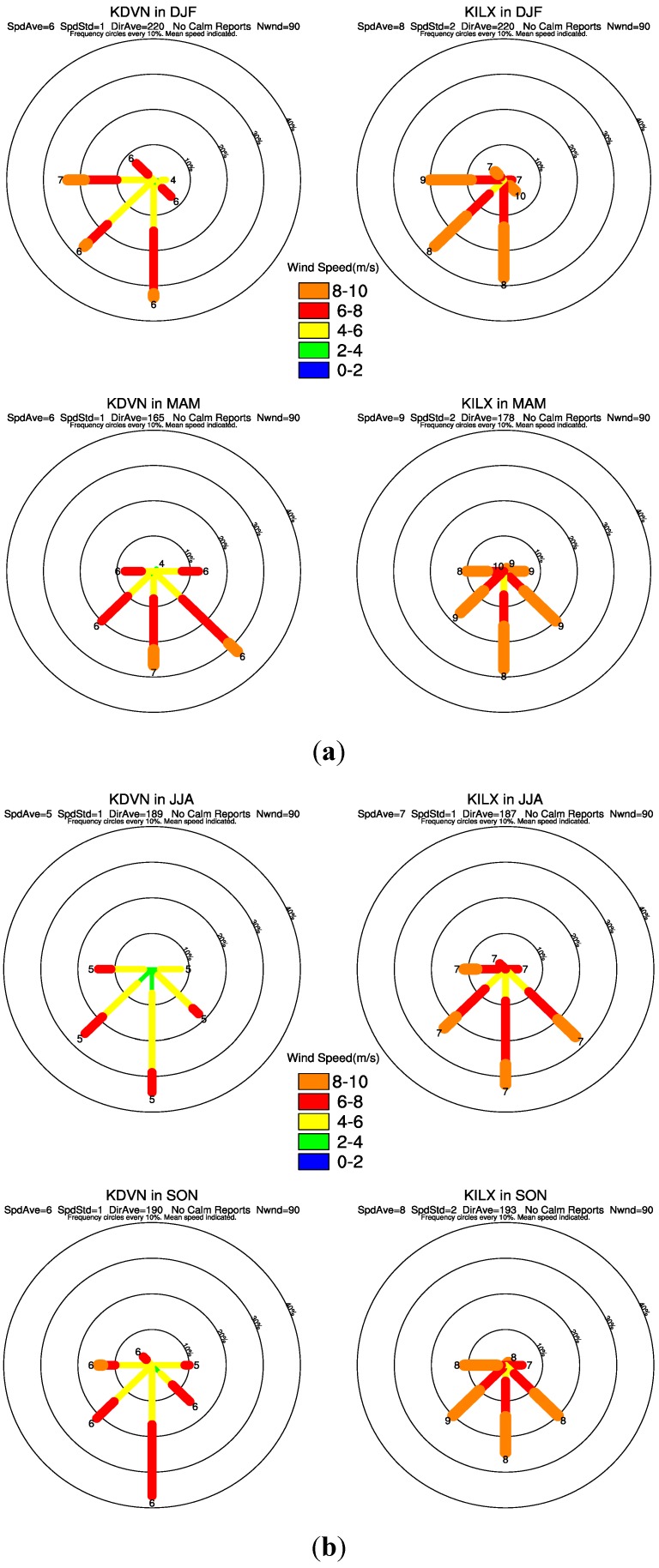
(**a**) Same as Figure 11 but for DJF (top) and MAM (bottom); (**b**) Same as Figure 11 but for JJA (top) and SON (bottom).

The ~80 m hub-height wind directions from each sounding station at 12:00 GMT are plotted in Figure 11 and Figure 12 as seasonal composite wind rose to assess any shift in the spatial coupling. In general, the two sounding data show similar distributions of wind direction although the wind speed may differ slightly. The annual sounding composites have the strongest speeds and greatest occurrence of wind direction from the south, southwest and southeast (Figure 11).

The positive LST anomalies (Figure 4a) are located primarily in the northern, northeastern and northwestern pixels near the wind farms, indicating a downwind warming effect, while the upwind pixels of the wind farms only show negative LST anomalies (Figure 4a). The sounding composites in MAM (Figure 12a) and SON (Figure 12b) show wind direction distributions similar to those in ANN. As expected, the positive LST anomalies are mostly located in the downwind directions (Figure 5b,d) while the upwind directions are often characterized by the negative LST anomalies (Figure 5b,d). JJA also has similar wind direction distributions (Figure 12b) as those in ANN but the downwind effect is much weaker in magnitude and located closer to the wind farms (Figure 5c), which may reflect the lower wind speed when compared to the other seasons. DJF has strongest wind speeds and its most prominent direction is from the west, southwest and south (Figure 12a). Consequently it has the most evident downwind warming effect, which is much more widespread than any other seasons (Figure 5a). Overall, the downwind effect is visible in all seasons and is proportional to the magnitude of the wind speed. 

We use the distance of ~2 km away from WFPs to minimize most impacts from turbulence downwind of the rotors (Figure 1c). This distance is for individual turbines but when the wind passes through an entire wind farm, the impact could last a greater distance. So it is likely that an entire wind farm would likely have residual impacts greater than 10 to 15 rotor diameters downstream.

We quantify this downwind warming effect (Table 1) by comparing the SCI values of the top 5%, 10% and 15% pixels with the strongest warming anomalies falling into UWFPs, DWFPs and NNWFPs compared with the corresponding SCI thresholds, 5.6%, 12.0%, and 16.0%, respectively. Note that the higher the SCI for DWFPs, the stronger the downwind effect. The SCI value ranges from 13.4% to 29.7% for DWFPs in all seasons, significantly higher than the 12.0% SCI threshold, suggesting the presence of a downwind wind farm effect. Generally DJF has the largest SCI value, followed by SON and MAM, and JJA has the smallest SCI value. In other words, the downwind warming effect is strongest in DJF, followed by SON, MAM and JJA, in an order that is consistent with our previous analyses of LST changes and wind speed. On the contrary, only one of the 12 SCI values for UWFPs and two of the 12 SCI values NNWFPs are larger than the corresponding SCI thresholds, indicating that it is very unlikely to have a strong warming LST anomaly seen over these two groups of pixels.

#### 3.3.5. Impacts of MODIS LST Uncertainties

The MODIS LST data represent the best quality retrieval possible from clear-sky conditions and have been widely used in many areas. However, they do contain errors and noise due to cloud and aerosol contamination and imperfection of retrieval algorithms [36], particularly at daily and small spatial scales. However, the spatial and temporal averaging applied here should largely remove such errors and uncertainties. The remaining residuals, if any, cannot accidentally create the spatial coupling between the warming and wind turbines shown above [17,18].

## 4. Conclusions

This study assesses impacts of three wind farms in northern Illinois using land surface temperature (LST) from MODIS for the period 2003–2013. Changes in LST between two periods (before and after construction of the wind turbines) and between wind farm pixels (WFPs) and nearby non-wind-farm pixels (NNWFPs) are quantified. We find a nighttime warming signal tightly spatially coupled with wind turbines in JJA and this warming signal is also apparent in other seasons as well. When examining interannual variability for the entire study period of 2003–2013, the areal mean nighttime LST of WFPs becomes prominently warmer than that of NNWFPs after the construction of wind farms. The estimated areal mean warming effect over the wind farms is found to be the largest in DJF (0.39 °C), followed by MAM (0.27 °C) and SON (0.26 °C), and smallest in JJA (0.18 °C). The annual mean warming effect is 0.26 °C. On the contrary, the daytime LST shows minimal or no impact of wind farms.

Analysis of seasonal variations in wind speed and direction from weather balloon sounding data and ASOS hourly observations from nearby stations suggests stronger winds correspond to seasons with greater warming and larger downwind impacts. The early morning soundings from two weather stations are representative of the nighttime boundary layer and exhibit strong temperature inversions across all seasons. The strong and relatively shallow inversion in JJA and SON leaves warm air readily available to be mixed down and spatially well coupled with the turbines. Although the warming effect is strongest in DJF and MAM, the spatial coupling is more spread out than in JJA and SON. It is estimated that most of the wind speeds at hub-height are within cut-in speed and therefore the turbine blades are always spinning and mixing the stably stratified air at nighttime. This supports the idea that the observed warming signal at nighttime, which is spatially coupled with the wind turbines, is likely due to downward transport of warmer air aloft to the surface by the turbulence caused by the spinning rotor blades.

Our results are generally consistent with Zhou *et al.* [17,18]. However, the wind turbine induced nighttime warming by 0.18–0.39 °C in our study region is smaller in magnitude and weaker in the spatial coupling with the wind turbines than the warming effect of 0.31–0.70 °C observed in Central-Western Texas [17,18]. In addition, Zhou *et al.* [17,18] showed the strongest warming effect and the best spatial coupling of the warming signal with the wind turbines in JJA while the weakest wind farm impact in DJF. Our results in the northern Illinois wind farms, however, have different seasonal variations of wind farm-induced nighttime warming effect. Several factors might help to explain the differences. First, the wind speed at nighttime is smaller in our study region than in Central-Western Texas. Also the seasonal variations in wind speed also differ between the two regions. Second, the total number and density of wind turbines in our study region is much lower than those in Central-Western Texas. Third, for ecosystems with more moisture and vegetation, the surface warms less because: (i) more energy is converted into latent heat via evapotranspiration and is transferred into ground heat storage and less energy for the increase of LST [37]; (ii) the nighttime inversion boundary layer is deeper and thus less sensitive to any forcing such as turbine-enhanced vertical mixing [38]. Further attribution of each factor’s individual contribution, however, is impossible based on analysis of satellite and meteorological observations. 

How wind farms modify hydrometeorology has been recently examined mostly in numerical studies, but detailed physical processes and spatial variations in wind farms-atmosphere interactions remain largely unknown and are currently under debate, due primarily to observational limitations and model deficiencies in describing these processes. Like any observations, remote sensed data have errors and uncertainties. However, remote sensing technology provides a new observational approach, as an emerging research topic, to detect, quantify and attribute wind farm impacts with spatial detail [17,18,19,20,21]. When combined with analyses of *in situ* observations, it may also help to uncover the physical mechanisms responsible for the satellite detected wind farm impacts.
